# Biochemical repercussions of light spectra on nitrogen metabolism in spinach (*Spinacia oleracea*) under a controlled environment

**DOI:** 10.3389/fpls.2023.1283730

**Published:** 2023-12-20

**Authors:** Moazzameh Ramezani, Dalton Thompson, Matte Moreno, Vijay Joshi

**Affiliations:** ^1^ Texas A&M AgriLife Research and Extension Center, Uvalde, TX, United States; ^2^ Department of Horticultural Sciences, Texas A&M University, College Station, TX, United States

**Keywords:** spinach, light spectrum, nitrogen, amino acids, minerals

## Abstract

**Introduction:**

Selecting appropriate light spectra of light-emitting diodes (LEDs) and optimal nutrient composition fertilizers has become integral to commercial controlled environment agriculture (CEA) platforms.

**Methods:**

This study explored the impact of three LED light regimes (BR: Blue17%, Green 4%, Red 63%, Far-Red 13% and infrared 3%, BGR; Blue 20%, Green 23%, Red 47%, Far-Red 8% and infrared 2%; and GR; Blue 25%, Green 41%, Red 32%, and Far-Red 2%) and nitrogen levels (3.6 and 14.3 mM N) on spinach (*Spinacea oleracea*).

**Results:**

Under limited nitrogen (3.6 mM), BGR light increased the fresh shoot (32%) and root (39%) biomass than BR, suggesting additional green light’s impact on assimilating photosynthates under suboptimal nitrogen availability. Reduced chlorophyll (a and b) and carotenoid accumulation, electron transport rate (ETR), and higher oxalates under limited nitrogen availability highlighted the adverse effects of red light (BR) on spinach productivity. Increased activities of nitrogen-associated enzymes (GOGAT; Glutamate synthase, GDH; NADH-Glutamate dehydrogenase, NR; Nitrate reductase, and GS; Glutamine synthetase) in spinach plants under BGR light further validated the significance of green light in nitrogen assimilation. Amino acid distributions remained unchanged across the light spectra, although limited nitrogen availability significantly decreased the percent distribution of glutamine and aspartic acid.

**Conclusion:**

Overall, this study demonstrated the favorable impacts of additional green light on spinach productivity, as demonstrated under BGR, than GR alone in response to nitrogen perturbation. However, the exact mechanisms underlying these impacts still need to be unveiled. Nevertheless, these outcomes provided new insights into our understanding of light spectra on spinach nitrogen metabolism.

## 1 Introduction

Since the onset of the coronavirus pandemic, the adoption rate of Controlled Environmental Agriculture (CEA) systems worldwide has been rising (Van Delden et al., 2021; Cowan et al., 2022; Zhang et al., 2022). Even though CEA-produced vegetables frequently carry price premiums, their share in fresh vegetable production will continue to expand due to increasing consumer demand for local food options. As an indoor food production system, CEA has offered a technological breakthrough to solve several problems associated with traditional farming and mitigate environmental and social challenges at the food-energy-water nexus (Lubna et al., 2022; Vatistas et al., 2022; Dsouza et al., 2023). Even if the CEA industry is booming, the science-based interventions to optimize the technical precision that identifies suitable light spectrums and nutrient management strategies to recover desired nutritional benefits need continual attention (Gómez et al., 2019; Sharathkumar et al., 2020; Neo et al., 2022).

Spinach is popular among vegetables due to its nutritional benefits, contributing to an estimated 40% of the leafy greens market (Batziakas et al., 2019; NASS, 2021). Although spinach accumulates many nutritional components, its accumulation is subjective to the growing environment. Maximizing the indoor production of spinach without compromising its nutrients is critical to its nutritional quality. Nitrogen (N) is an expensive input critical to maximizing plant productivity. Like most leafy greens, spinach requires excessive amounts of N fertilizers to produce higher biomass regardless of the production system (Markovic et al., 1988; Elia et al., 1998; Canali et al., 2014; Abdelraouf, 2016; Frerichs et al., 2022). Enhancing crop nitrogen use efficiency (NUE) without compromising quality and yield has become an apparent crop production strategy. Most higher plants reduce nitrate, the inorganic form of N, into an organic form, such as ammonia, by distinct enzymatic reactions initiated by nitrate reductase (NR) (Crawford, 1995). Ammonia is assimilated into amino acids glutamine and glutamate via individual isoenzymes of glutamine synthetase (GS), glutamate synthase (GOGAT), and glutamate dehydrogenase (GDH) (Ireland and Lea, 1999). Spinach’s nitrogen use efficiency (NUE) is poor due to the shallow root system (Stagnari et al., 2007; Marvi, 2009) and nitrate reduction efficiency (Neeteson and Carton, 2001; Koh et al., 2012). Although enhancing the NUE in spinach by exploiting existing genetic diversity has been proposed (Chan-Navarrete et al., 2014; Chan-Navarrete et al., 2016), N uptake is subjective to the production system. In production systems, where the supply of N or other nutrients is not limited, the uptake and assimilation of micronutrients or other phytochemicals are primarily subjected to manipulating environmental parameters.

Environmental optimization is crucial to improving the profitability of CEA platforms. As the CEA industry proliferates, manipulating the growing environment to maximize nutrient recovery needs continual research. Selecting appropriate light spectra of light-emitting diodes (LEDs) has become integral to commercial CEA platforms due to their flexibility in tailoring the light spectrum to maximize production (Balázs et al., 2022; Sheibani et al., 2023). Manipulating spectral quality has significantly impacted nutritional qualities among a wide range of high-value specialty crops (Hasan et al., 2017; Jones, 2018; Paradiso and Proietti, 2022). The different quantum efficiency and photoelectric conversion efficiencies of red and blue LED lights or their combinations result in varied energy consumption. Red and blue wavelengths impact photosynthetic performance(Johkan et al., 2010), morphogenesis (Dou et al., 2020; Chen et al., 2021), and metabolic composition (Kopsell et al., 2015; Trivellini et al., 2023) due to maximal absorption by chlorophyll a and b. However, the misconception of these spectra being the most efficient is challenged by several studies showing the positive impacts of green light on photosynthesis (Terashima et al., 2009; Hogewoning et al., 2012; Smith et al., 2017). As per the Emerson effect (Emerson et al., 1957), simultaneous exposure to red and far-red LED illumination enhances photosynthesis in plants, mainly morphological features such as leaf length (Li and Kubota, 2009) and yield or biomass (Kim et al., 2020). The higher absorbance of red and blue lights yields a higher quantum yield of CO_2_ assimilation (Q_Y_, moles of CO_2_ assimilated per mole of photons) than green light (Liu and Van Iersel, 2021).

On the other hand, despite its lower absorptance, green light can penetrate and excite chlorophyll deeper in leaves. Several studies have shown the significance of far-red light (700–800 nm) in mediating plant growth and developmental processes (Islam et al., 2014; Demotes-Mainard et al., 2016; Park and Runkle, 2017; Zhen and Bugbee, 2020). The interaction of these spectra on productivity is not always synergistic (Liu and Van Iersel, 2021) and is defined by the plant species, developmental stages, and growing conditions. Nevertheless, the impact of N or light spectrum on the productivity or N assimilation in spinach under indoor systems remains to be tested.

In the present study, we have evaluated the interaction of LED lights differing in the composition of ratios between blue, green, and red-light spectra under two N regimes to understand its impact on spinach performance in a soil-less media under a controlled growth chamber. Although each production system has unique challenges, using a soilless matrix facilitates uniform growth and control of nutrient media due to its inert chemical composition, allowing productivity assessment as a function of N or light applied. We have shown that supervised machine learning effectively predicts the root traits in a uniform soil-less matrix (Awika et al., 2021). The results of this work should serve as a reference for additional light optimization in commercial indoor spinach production and improve our understanding of the effect of light quality on N assimilation and biochemical attributes in spinach.

## 2 Materials and methods

### 2.1 Growth conditions

The experiment was performed in a controlled growth chamber at the Texas A&M AgriLife Research and Extension Center, Uvalde, Texas. The spinach variety ‘Space’ seeds were planted in a growth medium in pots (10.2 cm x 10.2 cm x 8.9 cm) containing Turface (Turface Athletics™ MVP, PROFILE Products LLC, Buffalo Grove, Illinois, USA).

Plants were grown under three light-emitting diodes (LED) lights (**Supplementary Figure 1**) (A) BR light (Red Bloom spectrum; Active Grow, Seattle, WA), which uses 17% Blue, 4% Green, 63% Red, 13% Far-Red and 3% infrared, (B) BGR light (Red Bloom Pro Spectrum, Active Grow, Seattle, WA) formulated with 20% Blue, 23% Green, 47% Red, 8% Far-Red and 2% infrared; and (C) GR light (White Pro Spectrum, Active Grow, Seattle, WA) formulated with 25% Blue, 41% Green, 32% Red, and 2% Far-Red spectra at the light intensity of 200 μmol m^−2^ s^−1^ biologically active radiation (400–800 nm) inside a growth chamber maintained under a 12/12 h light/dark cycle, 22°C, and 75% relative humidity.

After the seedling emergence, plants were fertilized with Peters^®^ professional ready mix (5-11-26, Everris NA Inc., Ohio, USA) every four days. Two concentrations of nitrogen - LN (3.6 mM) and HN (14.3 mM) were used for low and high N management. An additional N for the high N was provided using calcium nitrate, and equivalent calcium was compensated for the low N, as detailed earlier (Joshi et al., 2020; Awika et al., 2021).

### 2.2 Determination of biomass, minerals, and NUE

Fresh weight (mg) of root and leaves was measured at harvest (55 days after germination) using an analytical balance. The dry weight was measured by oven-drying at 70˚C for 72 h. The plant samples were analyzed for total N (TKN), NO_3_^−,^ and NH_4_^+^ using an EasyChem Plus analyzer (Chinchilla Scientific, Oak Brook, IL, USA). Total elemental analysis was conducted using ICP-OES (Thermo Scientific™ iCAP™ 7000 Plus Series, Waltham, MA, USA). The nitrogen use efficiency (NUE) percentage was calculated as a ratio of TKN x dry biomass (g)/N input (g) separately under HN and LN. For the rest of the minerals (Ca, Mg, K, P, Fe, Cu, Zn), nutrient utilization efficiencies (NuUtE) were calculated by taking the ratio of dry biomass per plant to mineral content using established methods (Corrado et al., 2021). Individual mineral use efficiencies were obtained by taking the ratio of individual mineral amounts recovered from plant tissue to the concentration (ppm) applied through fertilizers.

### 2.3 Amino acid extraction and quantification with UPLC-ESI-MS/MS

Approximately 10 mg lyophilized plant tissue samples were homogenized into a fine powder in a Harbil model 5G-HD paint shaker (Harbil, Wheeling, IL, USA) using 3 mm Demag stainless steel balls (Abbott Ball Company, CT, USA). Total free amino acids were extracted by suspending the homogenized samples in 100μL of 20mM cold HCl per mg of tissues, incubating on ice for around 20 minutes, and then centrifuging at a speed of 14,600xg for 20 min at 4°C. The extracts were filtered through a 96-well 0.45-μm-pore filter plate (Pall Life Sciences, USA). The filtrates were used for derivatization using AccQ•Tag3X Ultra-Fluor™ kit (Waters Corporation, Milford, MA, USA) as per the manufacturer’s protocol. L-Norvaline (TCI AMERICA, USA) was used as an internal control. Calibration curves were built using TargetLynxTM Application Manager (Waters Corporation, Milford, MA, USA). UPLC-ESI-MS/MS analysis was performed using Water’s Acquity H-class UPLC system equipped with Waters Xevo TQ mass spectrometer and electrospray ionization (ESI) probe. Water’s MassLynx™ software was used for instrument monitoring and data acquisition. The data integration and quantitation were conducted using Waters TargetLynx™ software.

### 2.4 Measurement of chlorophyll and carotenoid

The chlorophyll content (μmol of chlorophyll per m² of leaf surface) was measured using a portable chlorophyll content meter (MC-100, Apogee Instruments, Inc., Logan, UT, USA) from the fully expanded leaves of 6-week-old plants. Freeze-dried spinach tissue powders (10mg) were vortexed with 80% (v/v) acetone, sonicated (5 min, room temperature), and centrifuged (14,600×g, 5 min). The supernatant was used to measure absorbance at 470 nm, 645 nm, and 663 nm using a Multiskan GO microplate reader (Thermo Fisher Scientific, Waltham, MA, USA). Chlorophyll and carotenoids were determined using preestablished equations (Lichtenthaler and Wellbur, 1983). Chlorophyll fluorescence parameters were recorded using the portable fluorometer FluorPen 110 (Photon Systems Instruments, Czech Republic) after dark, adapting the leaves for 30 min with the leaf clips on fully expanded leaves of each plant. The F_o_ values represent the chlorophyll fluorescence emission associated with energy losses in the light-harvesting complexes of PSII (Kalaji et al., 2017), and F_m_ values show the maximum level of fluorescence from the dark-adapted leaves when all PSII reaction centers are “closed” with a saturating flash of light were measured from fully expanded spinach leaves and used to derive ratios (F_v_/F_m_; F_m_/F_0_; F_v_/F_0_). FT (instantaneous chlorophyll fluorescence) and Q_Y_ (quantum yield) were instant measurements. The polyphasic chlorophyll fluorescence (OJIP) transients were measured on fully developed leaves following a 20-minute dark adaptation. The PSII parameters obtained from the OJIP transient (F_o_ = F_30μs_, minimum fluorescence intensity; F_j_ = F_2ms_, fluorescence intensity at the Jstep; F_i_ = F_30ms_, fluorescence intensity at the I-step; F_p_ = maximum fluorescence intensity at the peak P of OJIP) were analyzed using Strasser method (Strasser et al., 2010). Stomatal conductance (gsw), photosynthetic electron transport rate (ETR), and PSII actual photochemical quantum yield (PhiPS2) were measured on a fully expanded leaf using LI-600 Porometer/Fluorometer (LI-COR Biosciences, Lincoln, NE, United States).

### 2.5 Enzyme activity and oxalate assays

The nitrate reductase (NR) activity was measured using the established method (Yaneva et al., 2002). Spinach tissue was homogenized in 100 mM phosphate buffer (pH 7.5) and then centrifuged at 14,600 x g for 15 min at 4°C. 100 uL supernatant extract was added to 200 μmol KNO_3_ and 0.2 μmol nicotinamide adenine dinucleotide, to estimate the NR activity. The reaction was stopped by adding 50 μL 1 m zinc acetate after 20 min incubation at 30°C. The mixture was centrifuged at 7600 x g for 5 min, and the absorbance was recorded at 540 nm using a MultiSkan Go microplate reader (Thermo Scientific, Waltham, MA, USA). One unit of NR activity was defined as the nitrite nitrogen produced content per gram of fresh weight per hour (μg g^-1^ h^-1^).

Glutamine Synthetase (GS) and Glutamate Synthase (GOGAT) activities were determined by extracting spinach tissue samples with 50 mM phosphate buffer (2 mM EDTA, 2 mM dithiothreitol, 1% insoluble polyvinylpyrrolidone, and 1.5% soluble casein, pH = 7.5) and centrifuged for 30 min at 12,600 x g. The GS and GOGAT activities were measured as per the established protocols (Cánovas et al., 1991). One unit activity of GS was expressed as mmol g-glutamylhydroxamate formed per gram per minute and GOGAT activity as mmol NADH oxidized per gram per minute.

NADH-Glutamate dehydrogenase (GDH) enzyme activity was measured using an established protocol (Robinson et al., 1991). One unit of GDH activity was expressed as oxidization or reduction of 1 µmol NADH per min.

The oxalate assay kit (Colorimetric) (ab196990) was used following the manufacturer’s instructions (Abcam, Cambridge, MA, USA) to detect oxalate levels in spinach leaves. The plant extracts were prepared by homogenizing 15 mg tissue, followed by incubation with assay buffer and centrifugation at 10,000 x g for 5 min. The reaction mixture was added and incubated for 30 min, and the optical density at 450 nm was measured using a MultiSkan Go microplate reader (Thermo Scientific, Waltham, MA, USA). The concentration of oxalate was then calculated from a standard curve.

### 2.6 Statistical analysis

The data presented corresponds to the mean value ± standard error. Descriptive and summary statistics, analysis of variance (ANOVA), and the principal component analysis (PCA) for various measurements were calculated using JMP 14.0.0 (SAS Institute, Cary, NC, USA). One-way and two-way ANOVA was also conducted where applicable, with α = 0.05 and significance set at p < 0.05. The significant differences among treatment groups were determined using the Turkey Kramer HSD at p = 0.05, and letter groupings were generated using a 5% significance level. The normality of the distribution was tested by a P-value < 0.05 in a Shapiro–Wilk test. The data was subjected to PCA and biplots to visualize general clustering, trends, and differences among samples for free amino acids and mineral contents.

## 3 Results

### 3.1 Effects of the light spectrum and nitrogen on biomass and NUE

**Figure 1:**
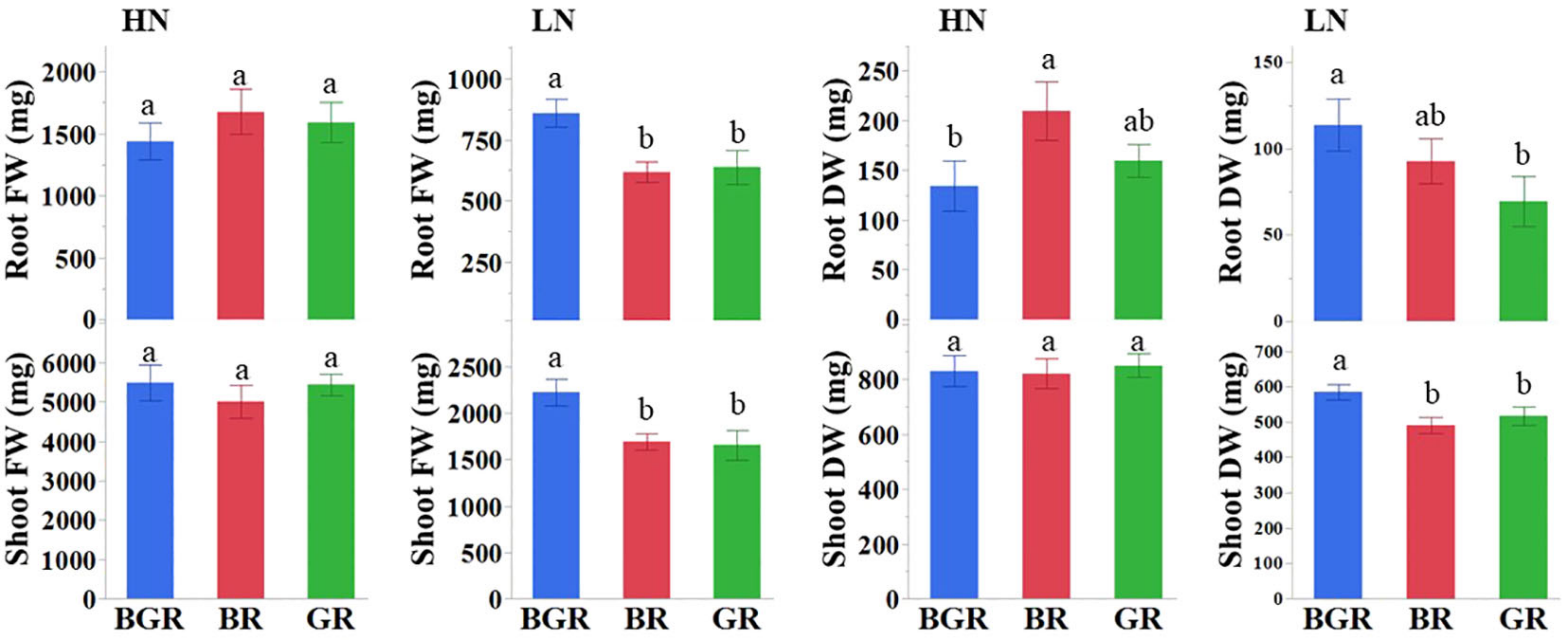
Effects of light treatments (BGR, BR, GR) on fresh (FW) and dry (DW) shoot and root biomass under high and low nitrogen. Different letters indicate statistically significant differences (P ≤ 0.05); N=10 plants.

We validated the impact of the nitrogen levels on the performance of spinach under three light spectrums by comparing the fresh and dry biomass and NUE using two N regimes. The Analysis of Variance (ANOVA) confirmed significant direct effects due to N for shoot and root biomass and the interaction effects due to N and light spectra only for dry root biomass (**Supplementary Table 1**). The shoot fresh biomass under three light spectra did not differ significantly under high N, although BGR-exposed plants had higher biomass at low N availability (**Figure 1**). The percentage increases in the fresh leaf biomass due to high N were comparable among BR (66%) and GR (61%) lights but were lower in the presence of BGR (41%) light (**Supplementary Table 2**). The NUE (g/g)^-1^ values based on shoot dry biomass across lights were comparable within the nitrogen treatment but were much higher in magnitude when the N was suboptimal (**Supplementary Table 2**). The analysis of root fresh weight revealed that BGR-exposed root biomass was higher (~35%) than other lights under limited N availability, with no significant differences among the light treatments under high N treatment. The root dry biomass under BR light was higher than BGR when N was not limiting. On the other hand, BGR-exposed plants under low nitrogen had higher root dry biomass than GR.

### 3.2 Interaction effects of nitrogen and light spectrum on chlorophyll accumulation and carotenoid content in spinach

**Figure 2:**
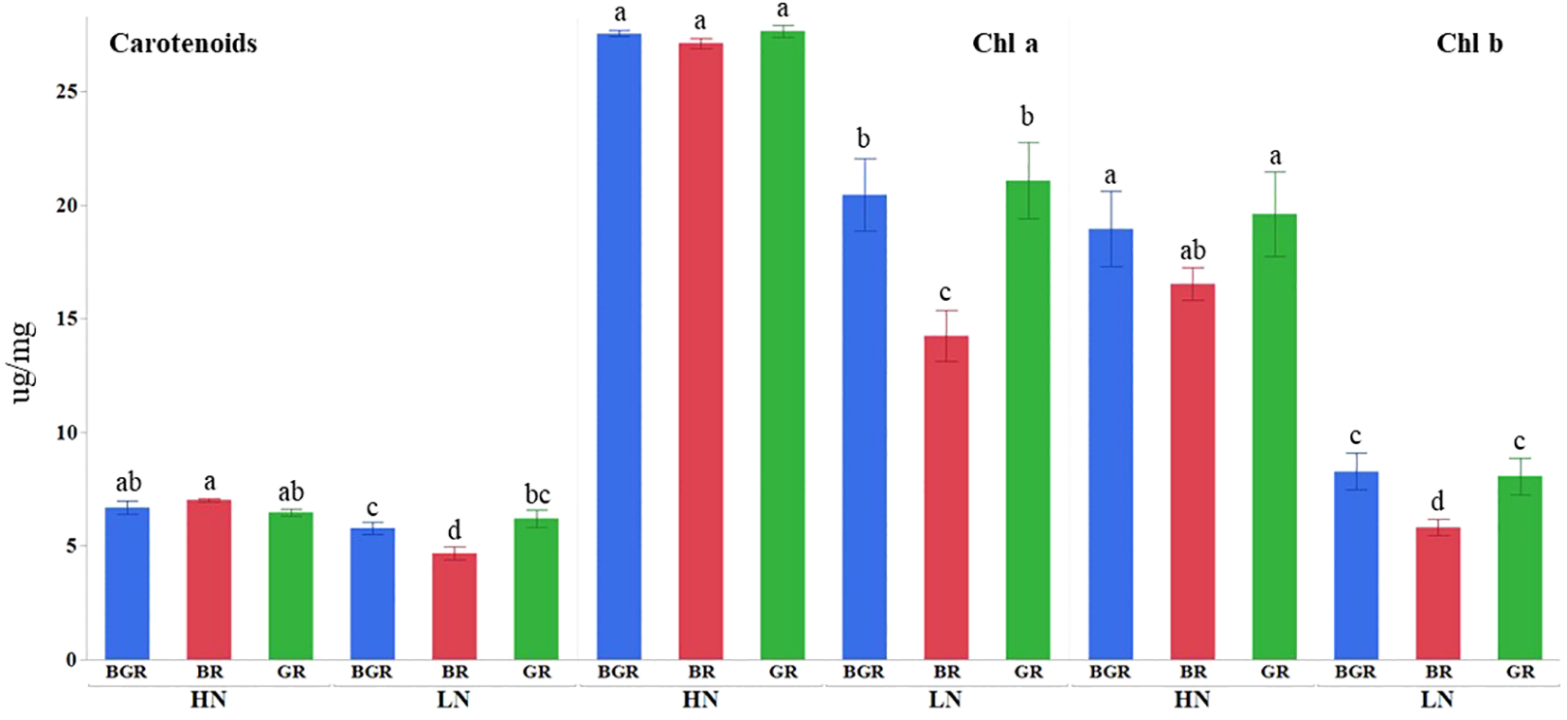
Effects of light treatments (BGR, BR, GR) and nitrogen levels (HN, LN) on chlorophyll (Chl a and Chl b) and carotenoid accumulation in spinach. Different letters indicate statistically significant differences (P ≤ 0.05); N=5.

**Figure 3:**
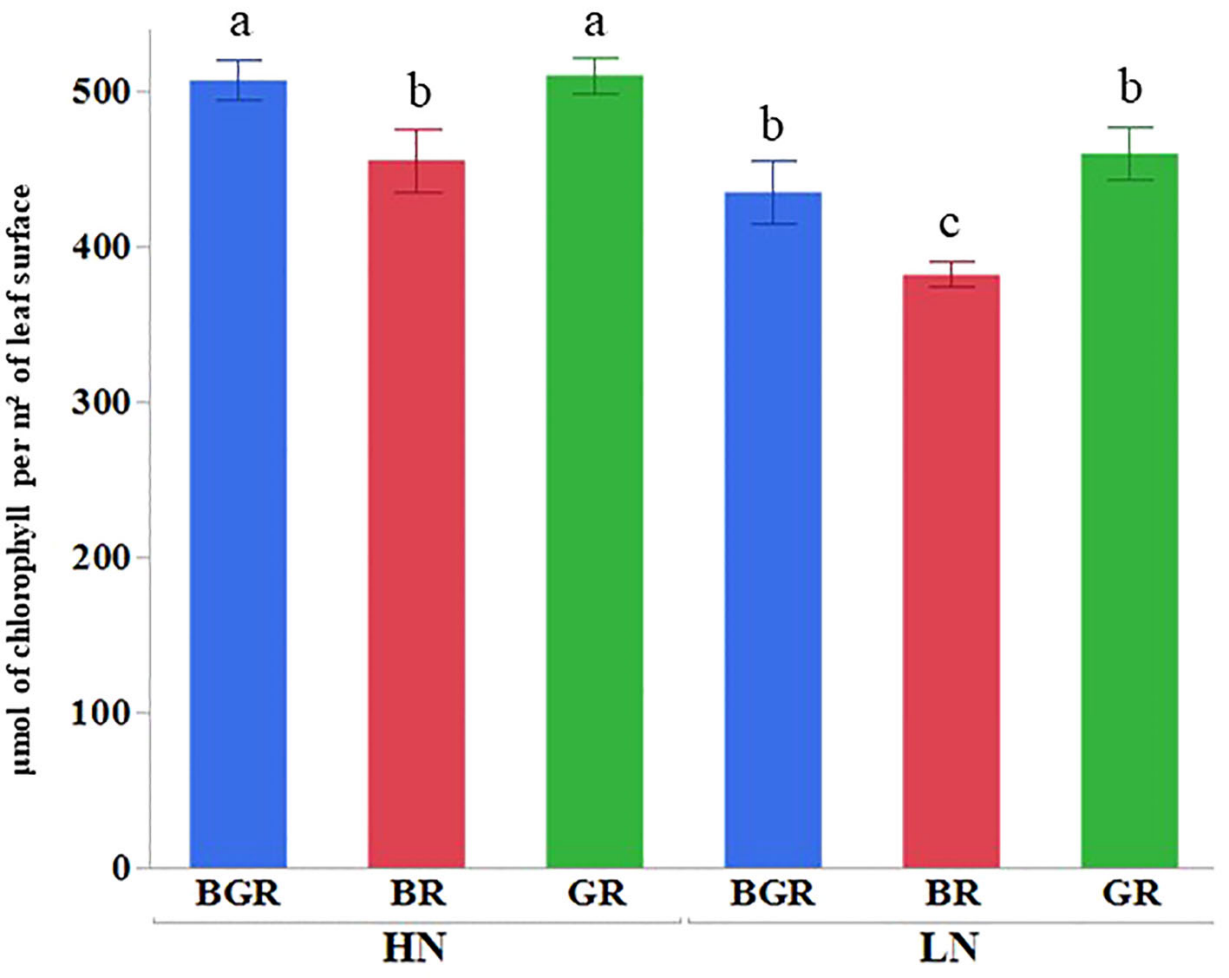
Chlorophyll (mol of chlorophyll per m² of leaf surface) content in spinach leaves under different lights (BGR, GR, BR) and nitrogen levels (HN, LN). Different letters indicate statistically significant differences (P ≤ 0.05); N=4.

Light spectra or their interaction with N significantly impacted the Chlorophyll a and carotenoid contents (**Supplementary Table 1**). High nitrogen enhanced Chl a, b, and carotenoid accumulation across lights (**Figure 2**) relative to low N. The Chl a and b content in BGR and GR light spectra under limited N was significantly higher (~46% and ~39%) than in BR lights. Similarly, the BR light decreased carotenoid accumulation by 27% under low N availability compared to BGR and GR. The chlorophyll (μmol of chlorophyll per m² of leaf surface) concentrations across lights showed significant changes in response to N and varying light spectra, but their interaction was insignificant (**Figure 3**; **Supplementary Table 1**). BR light plants accumulated significantly lower chlorophyll (11% under high N and 17% under low N) than the other two light spectra.

### 3.3 Chlorophyll fluorescence measurements

Chlorophyll fluorescence was recorded on fully expanded leaves of each plant using a FluorPen FP 110 (**Supplementary Figure 2**). F_0_ in the dark-adapted spinach leaves was lower in BGR and GR lights than in BR. No change in F_0_ readings was seen in response to the N status for all light treatments. Excepting BR light under both N regimes, F_m_ (maximum fluorescence) values were unchanged for BGR and GR lights. The F_v_/F_m_ and F_v_/F_0_ ratios were calculated to assess the efficiency of photochemical activities in PSII. The F_v_/F_m_ and F_v_/F_o_ did not differ significantly due to N treatments across lights. The effective quantum yield of photosystem (PS) II photochemistry (Q_Y_ and F_t_; instantaneous chlorophyll fluorescence) and OJIP analysis are often used as suitable markers for determining plant response to various stressors. Q_y_ or F_t_ values did not differ significantly across light spectra. When subjected to limited nitrogen availability, the plants exhibited higher F_t_ and lower Q_Y_ values under GR (**Supplementary Figure 3**).

**Figure 4:**
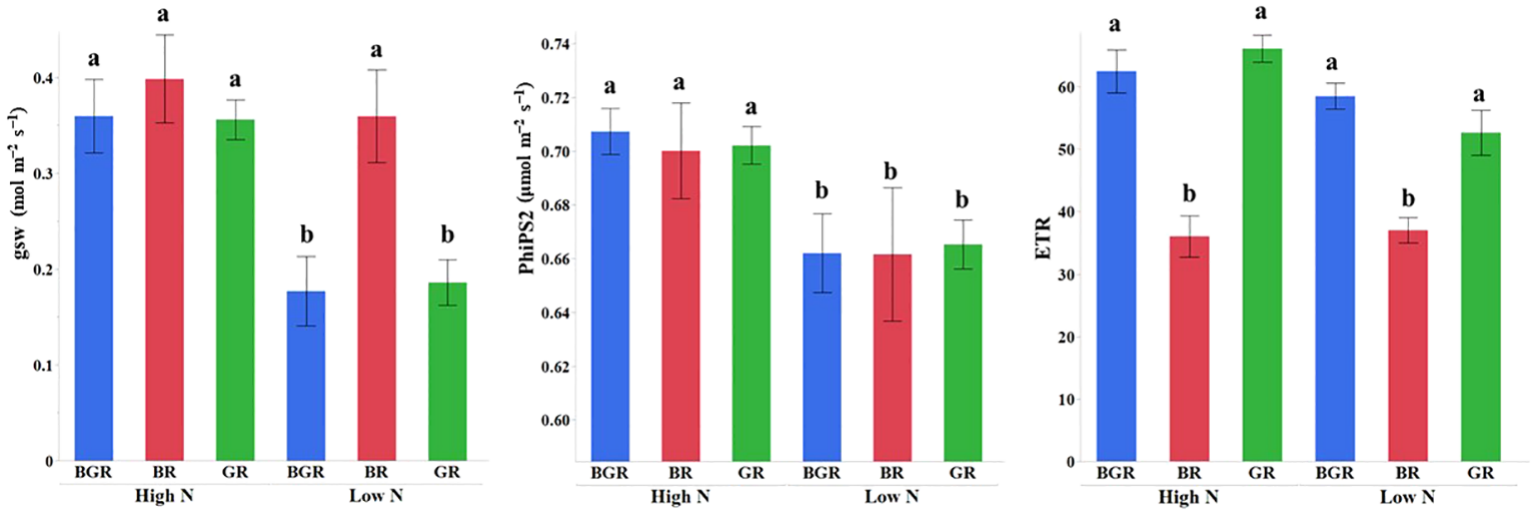
Effect of the light spectrum treatments (BGR, GR, BR) and nitrogen levels (HN, LN) on stomatal conductance (gsw, mol m^−2^ s^−1^), electron transport rate (ETR, μmol m^−2^ s^−1^), the quantum efficiency of PSII (PhiPS2). Different letters indicate statistically significant differences (P ≤0.05); N=4.

A significant interaction between light spectra and N was observed for the electron transport rate (ETR) and stomatal conductance (gsw) (p< 0.07) in spinach plants (**Figure 4**; **Supplementary Table 1**). Unlike unchanged conductance under BR light, a decrease in the stomatal conductance was recorded under BGR and GR with declining N content. In contrast, regardless of the N status, the electron transport rate (ETR, μmol m^−2^ s^−1^) remained the same under all lights, although it was significantly lower under BR. The quantum efficiency of PSII (PhiPS2) reduced with N availability but did not respond to spectral changes.

**Figure 5:**
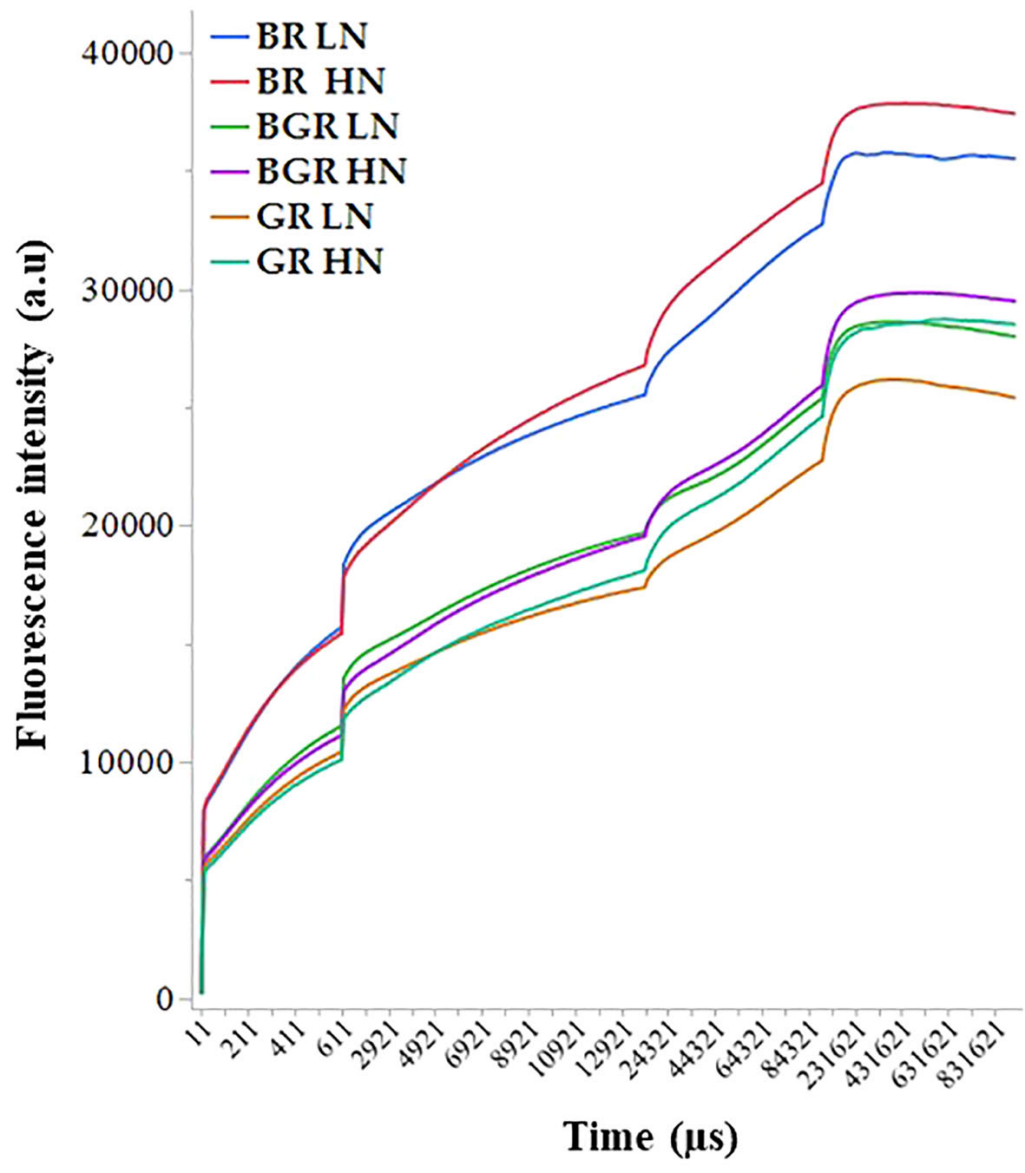
The intensity of Chlorophyll fluorescence during different steps of the OJIP curve exhibited by spinach leaves grown under varied light spectra (BGR, BR, GR) and nitrogen (HN and LN). Data was collected from four independent plants.

The non-destructive polyphasic OJIP chlorophyll fluorescence transients’ analysis under different light spectra was used to evaluate the photosynthetic function in the dark-adapted leaves. In all light spectra, plants growing under low N showed lower induction in Chl fluorescence intensity during all steps of the OJIP graph (F_0_, F_J_, F_I_, and F_m_) than high N. In contrast, Chl fluorescence intensity was consistently high in BR light during OJIP transitions (**Figure 5**).

### 3.4 Effects of light and N on activities of enzymes associated with nitrogen assimilation and oxalate contents

The analysis of variance (ANOVA) confirmed significant primary light x enzyme activities and nitrogen x enzyme activities interaction effects for all the enzymes (NR; Nitrate reductase, GDH; Glutamate dehydrogenase, GS; Glutamine synthase, GOGAT; Glutamate synthase) in the spinach leaves (**Supplementary Table 3**).

**Figure 6:**
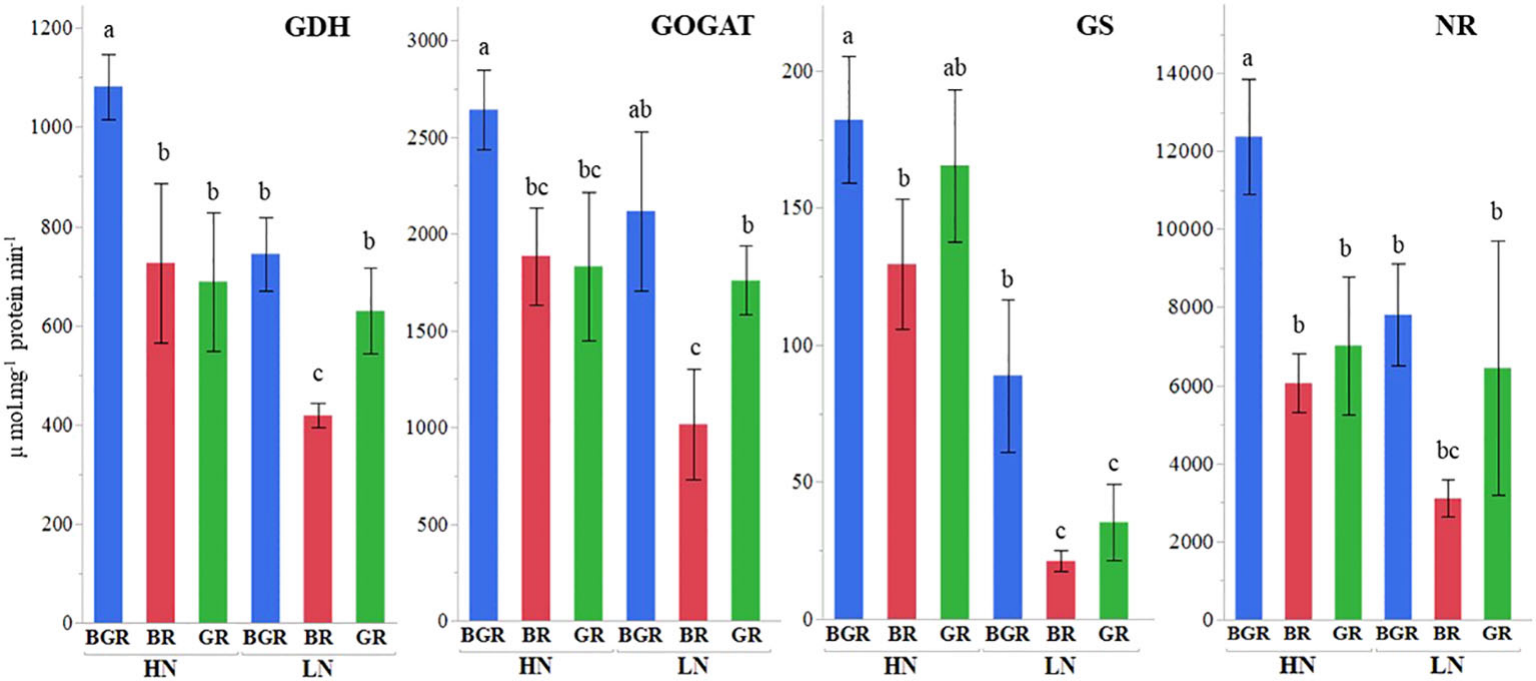
Effects of light spectra (BGR, BR, GR) and nitrogen levels (HN, LN) on nitrogen-associated enzymes GOGAT (Glutamate synthase), NR (Nitrate Reductase), GDH (Glutamate dehydrogenase), GS (Glutamine synthase), in the spinach leaf. Different letters indicate statistically significant differences (P ≤ 0.1). Each bar represents the mean of three replicates, and the error bars represent ± SE.

The GDH activity under BGR light was significantly higher under high N than BR and GR (**Figure 6**). GDH activity under limited N was significantly inhibited under BR light compared to BGR and GR. The GOGAT activity in spinach leaf was ~ 40% higher under the BGR light than under the GR and BR lights when N was not limiting. Unlike the BR light that decreased GOGAT under LN, the GOGAT activities under BGR and GR lights did not respond to N changes. GS activity significantly dropped under BR and GR relative to BGR lights when N was limiting. The NR activities showed no differences in response to N levels under BR or GR lights. The NR activity under BGR light was significantly higher than BR (104%) and GR (76%) when N was surplus.

**Figure 7:**
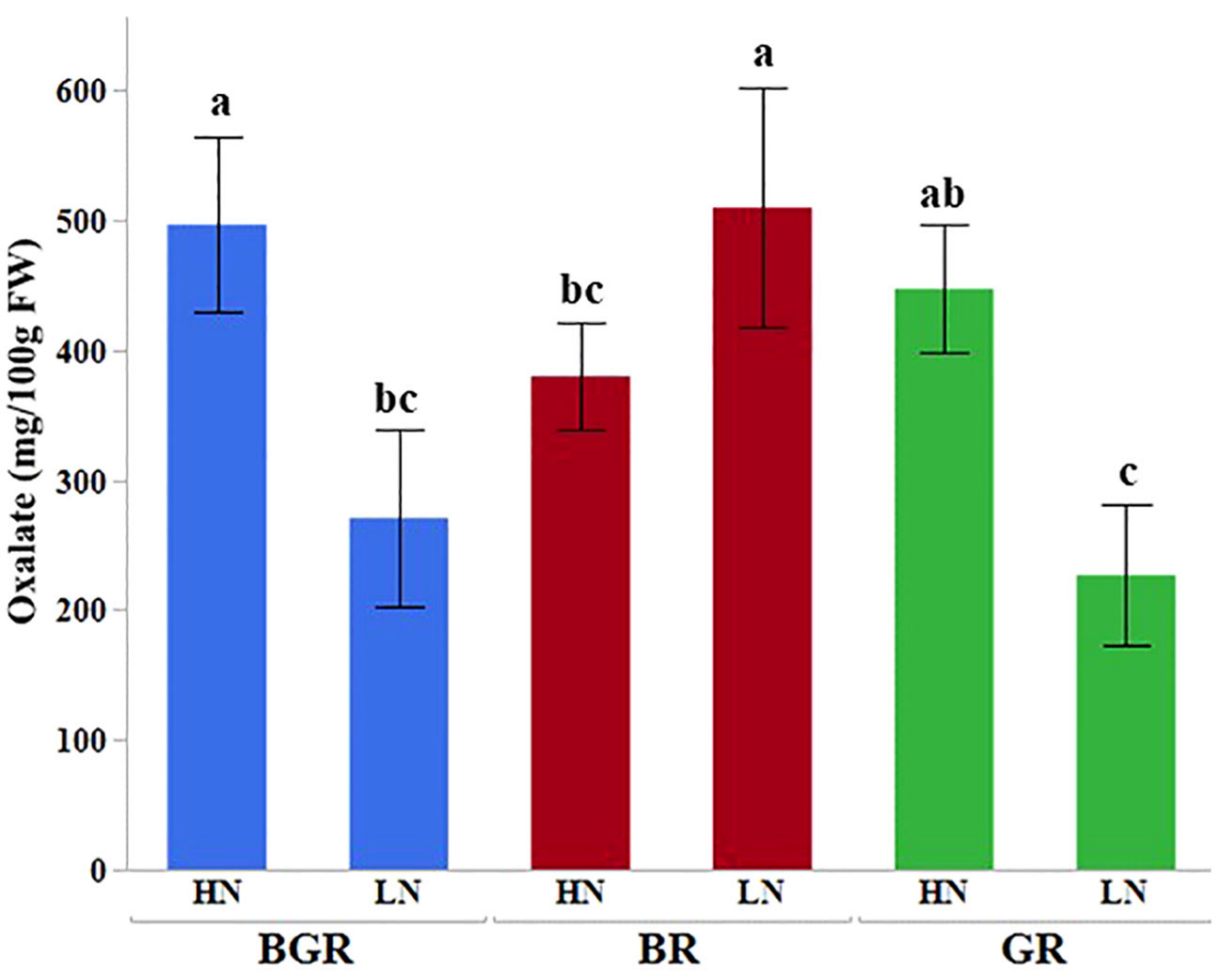
Effects of light (BGR, BR, GR) and nitrogen levels (HN, LN) on the oxalate content in the spinach. Each bar represents the mean of three replicates, and the error bars represent ± SE. Different letters indicate significant differences (P ≤0.05).

Oxalate content in spinach leaves did not differ significantly under different spectra when available N was excess. However, unlike BGR or GR lights, no significant change in the oxalate content was seen under BR light when N was limiting (**Figure 7**).

### 3.5 Accumulation of free amino acids in response to N and light spectra in spinach

**Figure 8:**
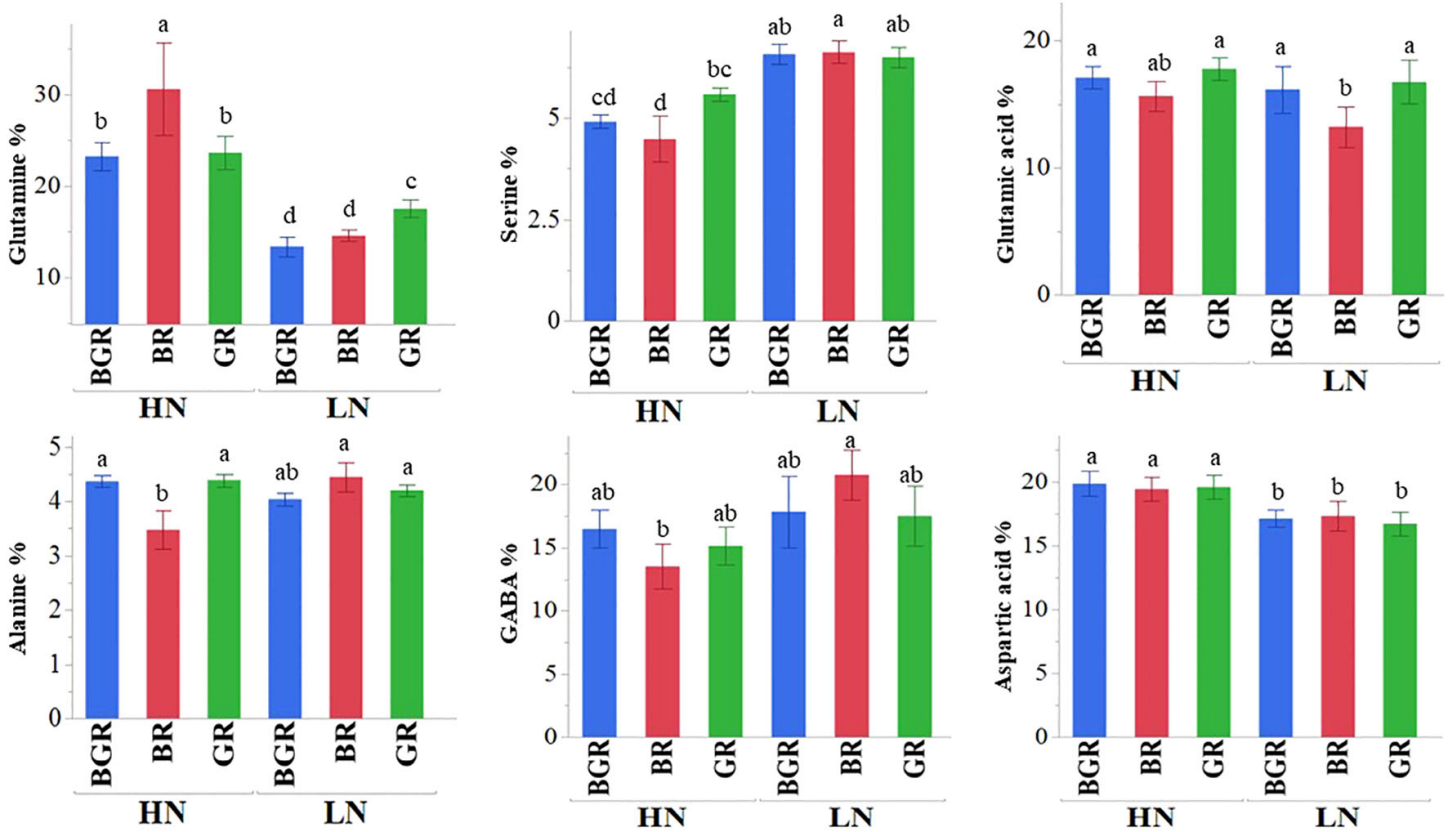
Changes in the percent accumulation of selective abundant free amino acids in spinach in leaf tissues under BGR, GR, BR lights, and nitrogen levels (HN, LN). Each bar represents the mean of six replicates ± SE. Different letters indicate significant differences (P ≤0.05).

Free amino acid accumulation and partitioning between leaf and root tissues across light treatments were evaluated in response to varied N levels. The analysis of variance (ANOVA) indicated that the percent distribution of most free amino acids showed significant differences between tissue type (leaf vs. root), light spectra, and N treatments (**Supplementary Table 4**) at p<0.1. Most amino acids differ significantly in response to N availability, tissue types, and their interaction. The three-way interaction between light, N, and tissue type was significant for asparagine, histidine, and alanine. Interaction between light and nitrogen was highly significant for less abundant amino acids (methionine, threonine) but also showed trends for N-rich abundant amino acids like asparagine and glutamine. Light alone significantly impacted histidine, phenylalanine, and alanine. Among the most abundant amino acids, glutamine and aspartic acid accumulation were enhanced under high N, irrespective of light spectra (**Figure 8**) in shoots. Under sufficient N availability, BR light accumulated significantly higher glutamine but lower serine and alanine in the shoot tissue. The percent distribution of glutamine, GABA, asparagine, aspartic acid, and alanine were higher in root tissue (**Supplementary Figure 4**). The percent distribution of the most abundant glutamine was significantly higher in the roots of BGR and GR-exposed plants than in BR under both N rates. The percent GABA distribution in the roots of plants under low N was significantly higher under high N in all respective light spectra.

**Figure 9:**
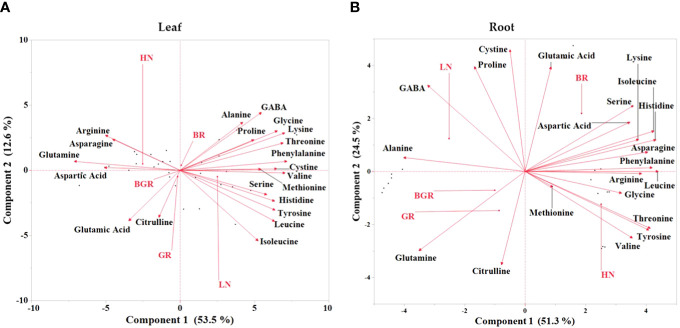
Principal component analysis (PCA) of free amino acids in spinach plant under different lights and nitrogen treatments in leaf **(A)** and root **(B)** tissues. Bi-plot for the first two principal components (PC) for free amino acids (scores) and treatments (loadings) as vectors for BGR, GR, BR lights and HN (High nitrogen) and LN (Low nitrogen).

To obtain a global overview of the effects of light spectra and nitrogen on the measured free amino acid pool sizes, we subjected the relative changes to Principal Component Analysis (PCA) (**Figure 9**). PCA projection demonstrated that the maximum variability in the data set differentiated between low and high nitrogen, with the first component (PC1) covering 54% of the data variance in shoot tissue. Both the principal components failed to discriminate between light spectra, suggesting their limited impact on the amino acid metabolome. Most N-rich amino acids, such as arginine, asparagine, glutamine, aspartic acid, and glutamic acid, were positively associated with HN in shoot tissue. While besides serine, GABA, and alanine, other amino acids were associated with LN in the PC1. The analysis of root data revealed that the largest source of variability in the dataset distinctly differentiated between nitrogen levels and light treatments. The first component (PC1) accounted for 52% of the variance in root tissue, emphasizing differentiation between low nitrogen and GR and BGR spectra while high nitrogen and BR spectra. Results showed an association between BR light and glutamic acid, serine, phenylalanine, and aspartic acid. On the other hand, root glutamine was associated with BGR and GR lights under limited N.

### 3.6 Effects of light and nitrogen on mineral accumulation and their Interactions

**Figure 10:**
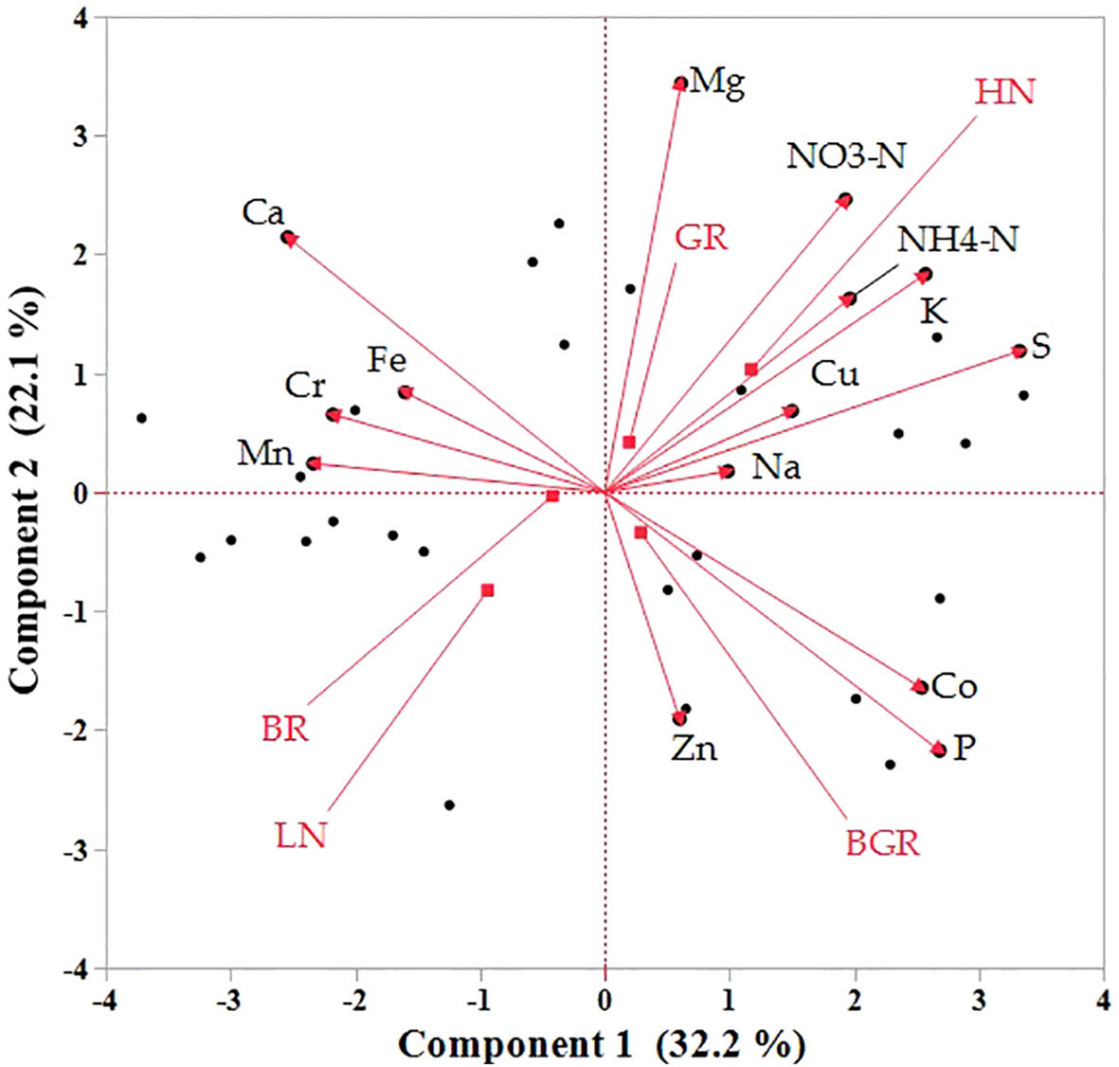
Principal component analysis (PCA) demonstrates the clustering of macro and microelements in response to N and light spectra into the first two principal components in the shoot. Bi-plot for the first two components (PC) for minerals(scores) and treatments (loadings) as vectors for BGR, BR, and GR lights and N levels (HN, LN).

The direct impact of the light spectrum and N availability on mineral absorption is not documented in spinach. Principal Component Analysis (PCA) was utilized to comprehensively overview the impacts of light spectra and nitrogen on the mineral accumulation and the relative changes (**Figure 10**). The Biplot graph PCA projection revealed that the most significant variations in the dataset distinctly distinguished between nitrogen levels and light. The first principal component (PC1) accounted for 32% of the variance in leaf tissue, highlighting the differentiation between nitrogen treatments. The high N was positively associated with TKN, NO_3_, HN_4_-N, K, and Cu.

In contrast, Ca, Se, Fe, and Cr were tightly associated with each other but negatively correlated with N-rich compounds. The light spectra have limited influence on the accumulation of macro or micronutrients. Most macro/micronutrient accumulation did not alter significantly across light spectra but was significantly higher for Total N (TKN nitrates (NO_3_) and magnesium in GR under limited N (**Supplementary Figure 5**).

## 4 Discussion

We investigated biochemical alterations in the nitrogen metabolism of spinach plants grown in varied light quality using blue and red light-emitting diodes (LEDs) along with a green light source. Due to their improved efficiency, LEDs are extensively used as supplemental lighting for indoor production of leafy greens and other horticultural crops. Specific narrow-banded light spectra within the electromagnetic spectrum affect the biochemistry and structure of photosystems, consequently influencing the nutritional content of the plants. Many physiological and molecular changes induced by specific light spectra and their interaction with nitrogen availability are poorly understood. Furthermore, with CEA poised to be prominent in commercial vegetable production, precise optimization of light quality to harness most nutraceutical benefits of leafy greens like spinach needs continual research.

Red and blue LED light or their combinations are popularly used for CEA production systems as leaves exhibit high absorbance and low reflectance in these spectral bands, promoting plant growth and development (Naznin et al., 2019). As the absorption of photons by chloroplasts near the adaxial surface induces heat dissipation of excess excitation energy with only little available to the ones in deeper leaf tissue (Sun et al., 1998; Nishio, 2000), it is suggested that blue and red photons are used less efficiently than green photons (Liu and Van Iersel, 2021). Several studies have underlined the importance of green light for uniform photosynthesis (Sun et al., 1998; Terashima et al., 2009; Hogewoning et al., 2012; Smith et al., 2017). In our study, we compared spectral composition that dominantly peaked for red light (red 63%, far-red; 13%, infrared; 3%) to that of two spectral distributions that prominently involved green light along with varied compositions of red and blue lights (**Supplementary Figure 1**). It has been argued that green light penetrates deeper into plant tissue than other colors to excite photosystems in deeper cell layers. In particular, measurements of chlorophyll fluorescence and other optical parameters within spinach leaves showed that light was absorbed in greater depths, with 50% of blue and green light reaching 125 and 240μm deep, respectively (Vogelmann and Evans, 2002) and blue light absorption by the lowermost chloroplasts was <5% of that of the uppermost (Evans and Vogelmann, 2003). Our results demonstrated that compared to BR (4% green light), incremental increases in the green light in BGR (23% green) and GR (41% green) lights enhanced Chl a and b accumulation by ~41% under limited N availability.

Further, the chlorophyll concentration in spinach leaves under BGR and GR lights was significantly higher than in BR, implying additive impacts of green light on chlorophyll synthesis. Although the quantum efficiency of PSII (PhiPS2) or Q_Y_ was unchanged under three spectra, the electron transport rates were significantly higher (77% under high N and 50% under low N) in BGR and GR than in BR alone. Green light contributes to photosynthesis more efficiently than red or blue light due to the non-photosynthetic absorption of green light by carotenoids (Mccree, 1971), which was confirmed in our experiment where carotenoids were significantly higher in BGR (23% green) and GR (41% green) and also showed higher shoot and root fresh and dry biomass under limited N availability. The shoot and root biomass under BGR were significantly higher than BR or GR when the N was limiting. The higher ETR and chlorophyll accumulation are consistent with studies that demonstrated deeper penetration of green light into leaves to assimilate CO_2_ efficiently (Brodersen and Vogelmann, 2010; Liu and Van Iersel, 2021).

Our results showed a significant reduction in stomatal conductance under low N availability in BGR and GR lights. It has been suggested that lower stomatal conductance could result from stomata responding directly to signals induced by N deprivation (Broadley et al., 2001). Intriguingly, stomatal conductance was not affected in BR (4% green) due to N on and was significantly higher than BGR (23% green) or GR (41% green) when N was limiting, which was consistent with studies in lettuce that showed the highest stomatal conductance under red and blue but lowest in the presence of green lamps (Kim et al., 2004). Green light has been reported to inhibit blue-light-induced stomatal opening (Talbott et al., 2002; Folta and Maruhnich, 2007; Matthews et al., 2020). We observed that the dry shoot and root biomass of plants grown in BGR were significantly higher than BR or GR under lower N. It is plausible to assume that supplemental green light in GR and supplemental red light in BR could negatively impact biomass under limited N. On the other hand, consistent with prior reports (Thi et al., 2020), additional green light in BGR compared to BR could have positively altered productivity and quality in spinach.

Frequent and substantial N applications are typical in traditional or indoor production systems. Optimization of N supply and its efficient management is critical to productivity, quality, and cost-prohibitive for indoor vegetable production. Increasing N supply enhances productivity and N content at the expense of NUE (Zhang et al., 2015; Frerichs et al., 2022). Although environmental groundwater contamination due to leaching under CEA is less of a concern, studies have shown that lowering N application increases NUE and reduces N losses (Song et al., 2009). The higher NUE under high or low N availability could result from saturated light in BGR or supplemental green light in GR compared to BR alone. Although research on the impact of different lights on NUE in spinach is still limited, higher NUE under the limited N we observed is consistent with other field-based or hydroponic studies in spinach (Canali et al., 2014; Zhang et al., 2015; Chan-Navarrete et al., 2016). Nevertheless, either a positive role of blue light on N allocation from root to shoot, only under limited nitrogen (Liang et al., 2022) or no significant impact on N uptake or utilization due to red or blue lights or their combinations (Clavijo-Herrera et al., 2018; Pennisi et al., 2019a; Pennisi et al., 2019b) has also been shown in lettuce.

In the present study, all light spectra demonstrated typical OJIP phases, indicating that the plants were photosynthetically active. Our results confirmed that nitrogen-induced changes in OJIP parameters are susceptible to any change in PSII activity, where light spectra efficiently impacted PSII activity in nitrogen stress. The OJIP curve shows changes associated with reducing the primary electron acceptor of photosystem II (PSII) and the efficiency of electron transport. The spectral composition alters the photosynthetic efficiency, the distribution of excitation energy between PSI and PSII, and the balance between photochemical and non-photochemical quenching mechanisms, leading to variations in the amplitude, kinetics, and shape of the OJIP curve. Unlike GR or BGR, higher fluorescence intensity in BR suggests higher absorption of red light by chlorophyll a and b, promoting the excitation of electrons in PSI and PSII, leading to a distinct OJIP curve shape with rapid transitions between the O, J, I, and P steps. Rapid transitions induced in the OJIP curves due to red light are consistent with studies in other species (Costa et al., 2021).

The data demonstrated that the most predominant amino acids in spinach leaves were major N-rich transporting amino acids such as glutamine, glutamic acid, aspartic acid, GABA, alanine, and serine. Among the major amino acids, the percent accumulation of glutamine and aspartic acid was significantly reduced under limited N. On the other hand, excepting alanine, GABA, and serine under BR, the percent accumulation of most amino acids remained the same irrespective of N treatment under all lights. As demonstrated by PCA analysis, most N-rich amino acids were clustered with high N in leaf tissue but did not respond to the light spectrum significantly, signifying light-induced changes in their accumulation were less pronounced in spinach leaves. Intriguingly, the negative association of glutamic acid, aspartic acid, and BR with glutamine, BGR, and GR in root tissues implicates the role of the light spectrum in the partitioning and assimilation of amino acids. Unlike reports in lettuce (Bian et al., 2018), adding green light to continuous red and blue light did not significantly alter the nitrate or total N content in spinach under sufficient nitrogen but showed an increase when N was limited. Consistent with other studies (Bian et al., 2018), we speculate that the additional green light in BGR, along with relative contributions of blue and red light, may have collectively resulted in enhancing NR and GDH activities under high N and GDH, GOGAT and GS activities compared to BR, under limited N.

The varied mineral composition under different light spectra in our data was consistent with other studies using white, red, and blue lights in spinach (Thi et al., 2020) and red, blue, and green lights in lettuce (Razzak et al., 2022) and microgreens (Kamal et al., 2020) or red and blue (Brazaitytė et al., 2021) in microgreens. Iron accumulation in spinach was also reported to be affected by its concentration in hydroponic solution rather than by the ratio of red to blue lights (Vaštakaitė-Kairienė et al., 2022). The positive association between nitrogen and oxalate in spinach was in agreement with several other studies (Ota and Kagawa, 1996; Zhang et al., 2005; Solberg et al., 2015; Joshi et al., 2021). Although similar to other reports (Gao et al., 2020), the oxalates under BR were significantly lower than BGR under high N, and unaffected oxalate accumulation under low N in BR suggests the possibility of N-independent regulation of its synthesis in spinach.

## 5 Conclusion

Taken together, the research underscores the dynamic interplay between light quality and nitrogen availability in modulating spinach productivity and quality. The shoot biomass of spinach was least influenced by additional green light but showed significant increases in the root fresh biomass when the nitrogen was limited. The impacts of green light (GR and BGR) showed additive effects based on higher chlorophyll concentrations, electron transport rates, and higher activities of nitrogen assimilatory enzymes (GDH, GOGAT, GS, and NR). The positive impacts of green light were more apparent when nitrogen was limited, based on higher accumulation of chlorophylls (a and b) and percent accumulation of glutamic acids or glutamine. This study provides new insights into regulating biochemical and physiological aspects under different light spectra in spinach. Additional research involving global expression analysis and manipulation of the relative contribution of light intensity and spectral distribution would help elucidate unknown nitrogen-associated mechanisms.

## Data availability statement

The original contributions presented in the study are included in the article/**Supplementary Material**. Further inquiries can be directed to the corresponding author.

## Author contributions

MR: Formal analysis, Investigation, Methodology, Software, Writing – original draft, Data curation. DT: Data curation, Formal analysis, Methodology, Writing – original draft. MM: Formal analysis, Methodology, Writing – original draft, Investigation. VJ: Formal analysis, Investigation, Methodology, Writing – original draft, Conceptualization, Funding acquisition, Project administration, Resources, Software, Supervision, Visualization, Writing – review & editing.
