# Supplementary figures and images for: Biochemical repercussions of light spectra on nitrogen metabolism in spinach (Spinacia oleracea) under a controlled environment
Source: Front Plant Sci. 2023 Dec 20;14:1283730. doi: 10.3389/fpls.2023.1283730 (PMC10765523; doi:10.3389/fpls.2023.1283730)

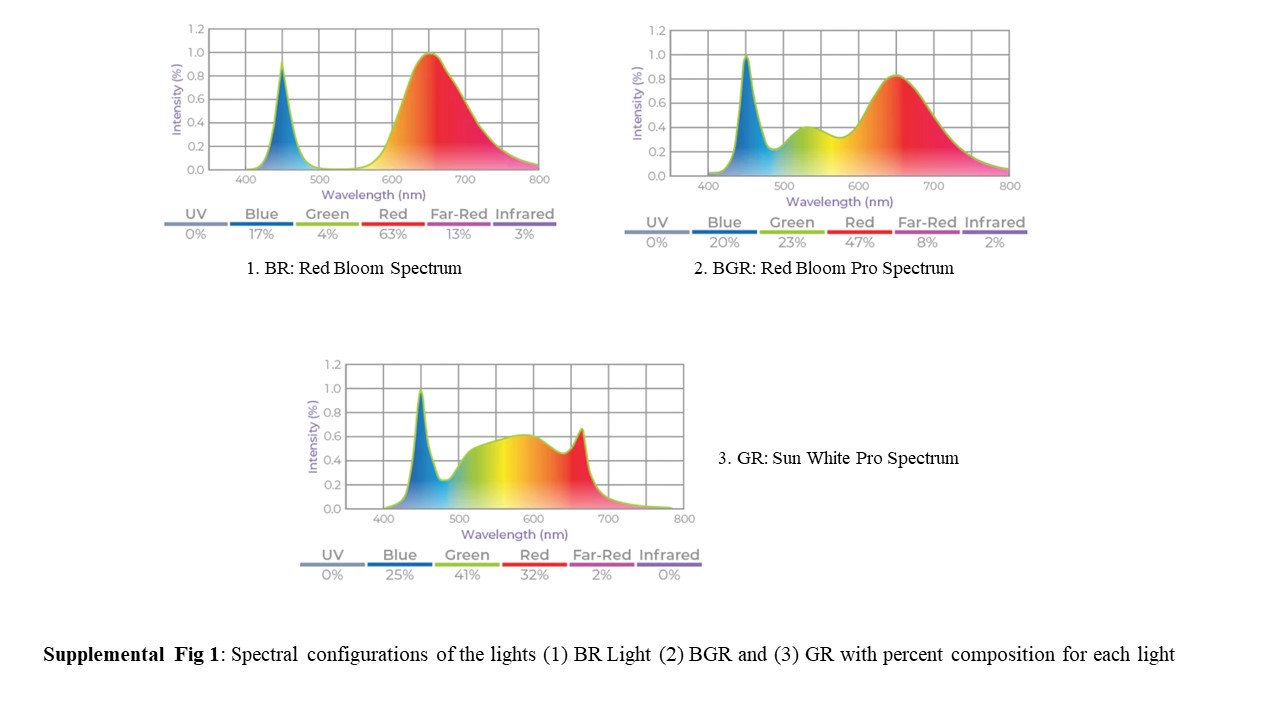

Supplement: Supplementary file 1 [file Image_1.jpeg]

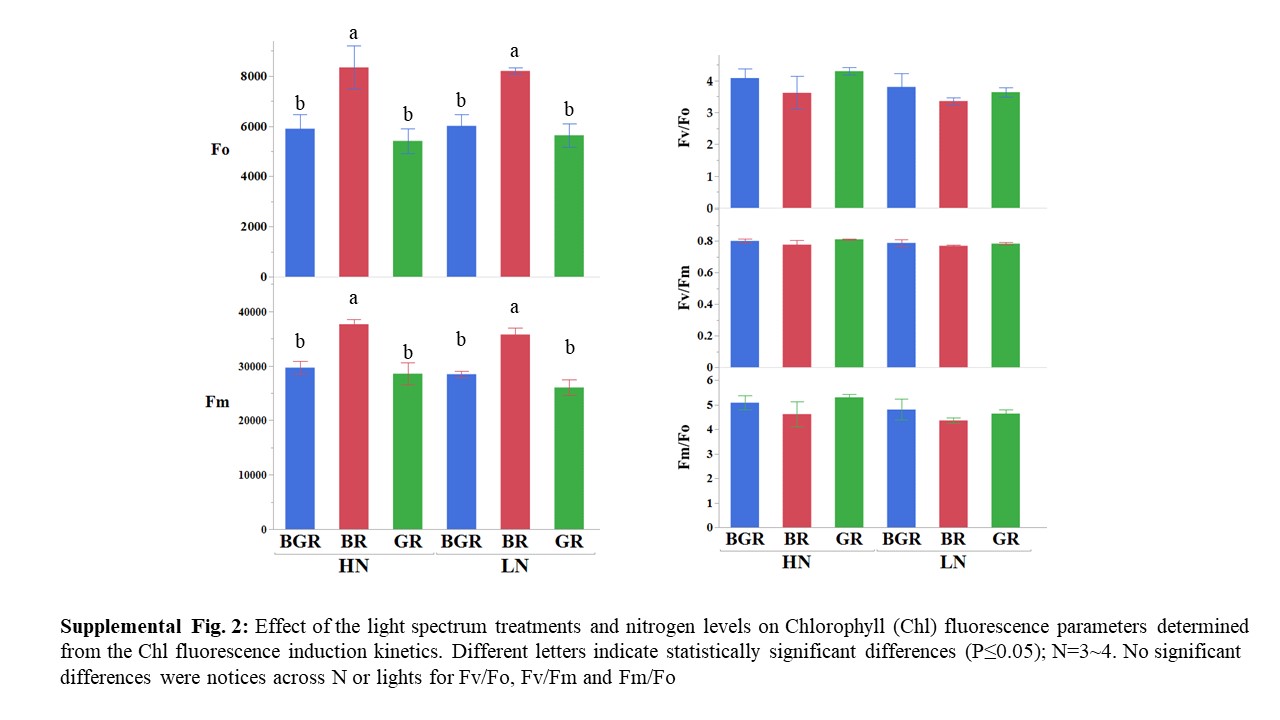

Supplement: Supplementary file 2 [file Image_2.jpeg]

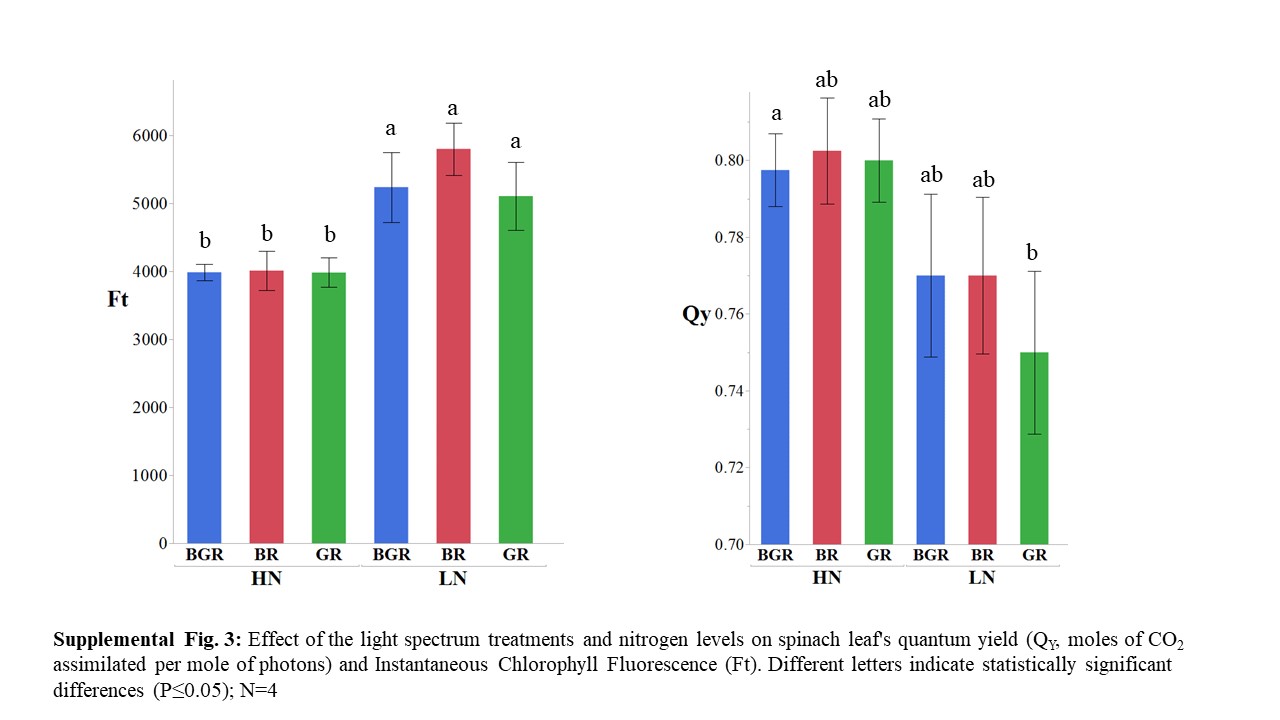

Supplement: Supplementary file 3 [file Image_3.jpeg]

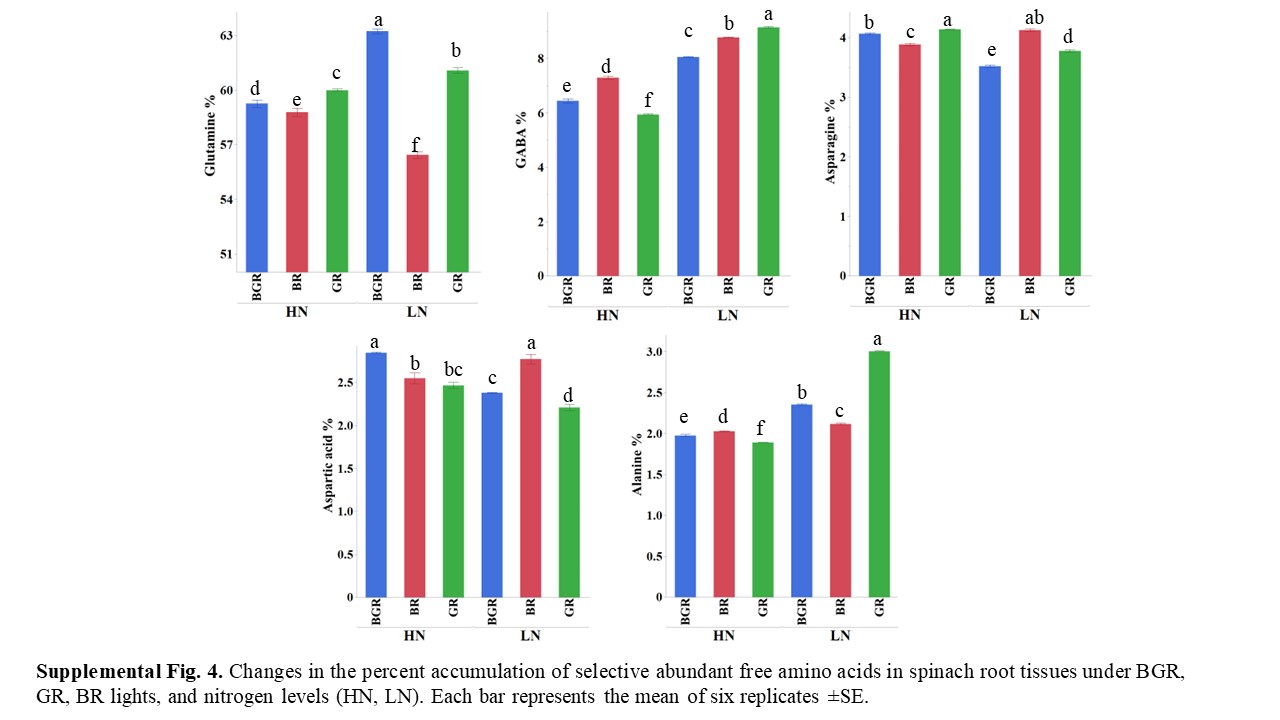

Supplement: Supplementary file 4 [file Image_4.jpeg]

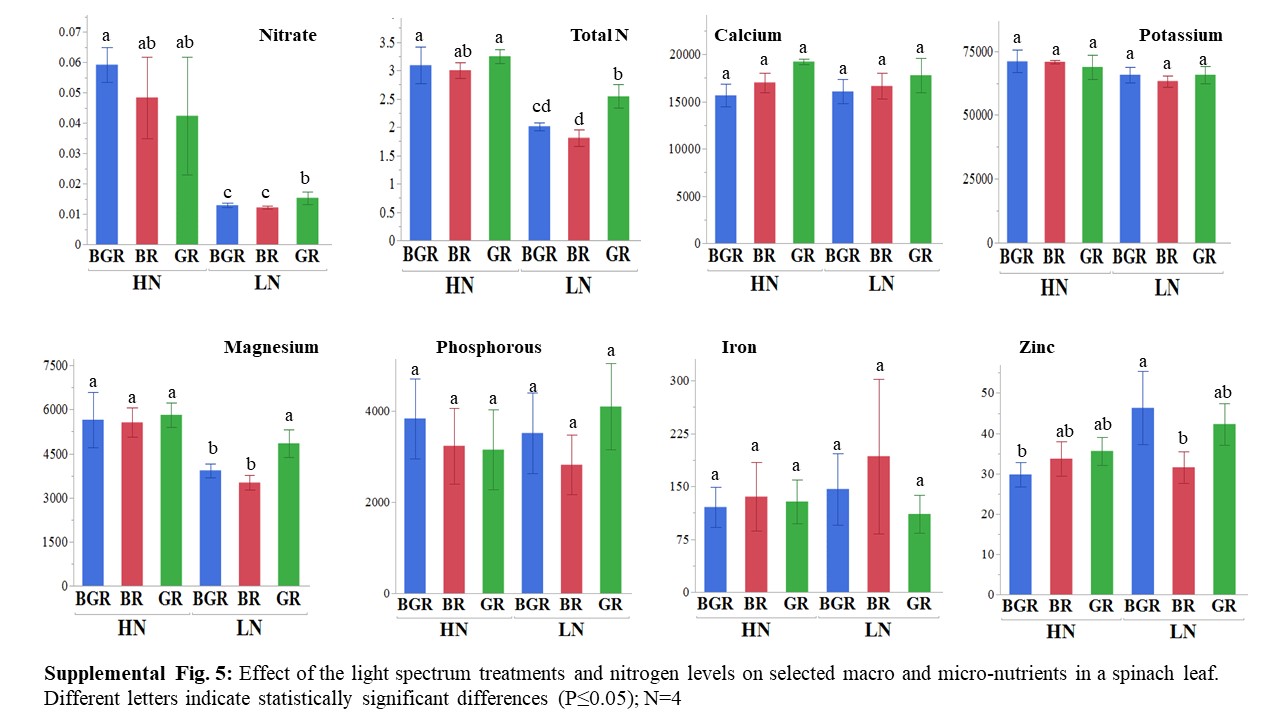

Supplement: Supplementary file 5 [file Image_5.jpeg]
